# The evolution of symptoms of nervous system dysfunction in a First Nation community with a history of mercury exposure: a longitudinal study

**DOI:** 10.1186/s12940-024-01089-9

**Published:** 2024-05-31

**Authors:** Aline Philibert, Judy Da Silva, Myriam Fillion, Donna Mergler

**Affiliations:** 1 Centre de recherche interdisciplinaire sur le bien-être, la santé, la société et l’environnement (CINBIOSE), Université du Québec À Montréal, CP 8888, Succ. Centreville, Montréal, Québec H3C 3P8 Canada; 2 Grassy Narrows First Nation, General Delivery, Grassy Narrows, ON P0X 1B0 Canada; 3 Département Science et Technologie, Université TÉLUQ, 5800 Saint-Denis St, Montréal, Québec H2S 3L4 Canada; 4 Département des sciences biologiques et CINBIOSE, Université du Québec À Montréal, CP 8888, Succ. Centreville, Montréal, Québec H3C 3P8 Canada

**Keywords:** Mercury, Past exposure, First Nation, Nervous system dysfunction, Symptom clusters, Progression of neurological symptoms, Longitudinal mixed effects models

## Abstract

**Background:**

Since the 1960’s, mercury (Hg) contamination of the aquatic environment of Asubpeeschoseewagong Anishinabek (Grassy Narrows First Nation) territories has impacted the community members’ traditions, culture, livelihood, diet and health. Despite decreasing Hg exposure over time, a recent study suggested that long-term exposure contributed to later-life symptom clusters of nervous system dysfunction. Here, the objective was to evaluate, 5 years later, the prevalence and progression of these symptoms and examine the contribution of long-term, past Hg exposure.

**Methods:**

The symptom questionnaire, applied in the 2016/17 Grassy Narrows Community Health Assessment (GN-CHA) (Time 1), was re-administered in the 2021/22 Niibin study (Time 2). A total of 85 adults (median age: 47y; range: 29-75y) responded at both times. Paired statistics were used to test the differences (Time 2 – Time 1) in self-reported symptom frequencies. The symptom clustering algorithm, derived from the entire study group of the GN-CHA (*n* = 391), which had yielded 6 clusters, was applied at Time 1 and 2. Equivalent hair Hg measurements (HHg) between 1970 and 1997 were used in Longitudinal Mixed Effects Models (LMEM), with a sub-group with ≥ 10 repeated HHg mesurements (age > 40y), to examine its associations with symptom cluster scores and their progression.

**Results:**

For most symptoms, paired analyses (Time 2 – Time 1) showed a significant increase in persons reporting “ very often” or “all the time”, and in the mean Likert scores for younger and older participants (< and ≥ 50y). The increase in cluster scores was not associated with age or sex, except for sensory impairment where a greater increase in symptom frequency was observed for younger persons. LMEM showed that, for the sub-group, long-term past Hg exposure was associated with most cluster scores at both times, and importantly, for all clusters, with their rate of increase over time (Time 2 – Time 1).

**Conclusions:**

The persistence of reported symptoms and their increase in frequency over the short 5-year period underline the need for adequate health care services. Results of the sub-group of persons > 40y, whose HHg reflects exposure over the 28-year sampling period, suggest that there may be a progressive impact of Hg on nervous system dysfunction.

**Supplementary Information:**

The online version contains supplementary material available at 10.1186/s12940-024-01089-9.

## Background

Symptoms are experienced sensations and bodily changes which, for many people, constitute the basis for seeking health care [1]. Patient-reported symptoms serve to initiate the process of differential diagnosis and follow-up treatment [2]. For neurodegenerative and neuroinflammatory diseases, symptoms can be focused and/or widespread depending upon the brain structures involved and the severity of the disorder. Symptoms of mercury (Hg)-related nervous system dysfunction pose a particular challenge since symptoms may not reflect current exposure, but can result from past exposures [3, 4]. Differential diagnoses depend strongly upon physicians’ knowledge of the history of exposure [5].

Much of our knowledge on symptoms of Hg poisoning comes from the decades of studies carried out with the victims of the Minamata disaster, the first large-scale poisoning associated with Hg contaminated fish and shellfish consumption [6–12]. Between 1932 and 1968, waste-water containing methyl-Hg (MeHg) chloride, was discharged by a chemical factory into Minamata Bay, Japan, exposing thousands of fish-consumers [6, 13]. Sentinel symptoms of Minamata Disease include muscle cramps, four-limb numbness, tendency for stumbling, difficulty in fine finger task, and limited peripheral vision [6, 8, 14, 15]. The neurological examination for Minamata Disease certification, carried out in 2009, included frequency and onset of symptoms [3]. Analyses of 973 claimants from the Minamata region revealed that for 65%, the first symptom occurred 10 years after the discharge was halted; more symptoms appeared in the subsequent years. There was a positive correlation between overall symptom frequency score and year of onset, suggesting a worsening of the severity of symptoms over time [3].

In Canada, between 1962 and 1975, a chloralkali plant discharged over 9 metric tons of inorganic Hg into a river that flowed into the traditional territories of Asubpeeschoseewagong Anishinaabek (also known as Grassy Narrows First Nation), located in Northern Ontario [16]. Hg, methylated in the aquatic system, was bio-accumulated and bio-magnified in fish [17], attaining concentrations 50 times the reference guidelines for fish consumption [18, 19]. For the people of Grassy Narrows First Nation, fish was central to their culture and livelihood, as well as their dietary mainstay [20]. Fish Hg concentrations began to decrease after emission controls were ordered in 1970 and, in 1975, the plant installed a non-Hg-based process. Levels stabilized around 1985 [19], but current levels remain high, possibly through ongoing erosion of high Hg particles by the river, as it meanders through contaminated floodplains [21]. Between 1970 and 1997, hair and blood samples were taken by governmental biomonitoring programs to assess Hg exposure of this First Nation community [22]. Biomarkers of Hg exposure, paralleled fish Hg concentrations [4, 23].

Since the disaster, Dr. Masazumi Harada and colleagues have carried out a series of examinations of people from Grassy Narrows, using the Japanese protocol for Minamata Disease [24]. At their first visit in 1975, despite very high concentrations of hair Hg (one third were above 30 µg/g, with a maximum of 80 µg/g), the examinees showed mild symptoms of Minamata Disease [25]. When the team returned in 2002/2004, all examinees, who had hair Hg concentrations ≥ 50 µg/g in 1975, were deceased [25]. Among the 44 people that were examined and for whom hair Hg was assessed, although mean hair Hg levels were low (2.07 µg/g ± 2.87), over 50% received a diagnosis of Minamata Disease or Minamata Disease with complications [25, 26]. Since hair Hg concentrations at the time of examination were low, the authors raised the question of the impact of long-term exposure to Hg, coupled to aging and other possible conditions [25].

In 2016, Grassy Narrows First Nation initiated a comprehensive house-to-house survey of their health and well-being, the Grassy Narrows Community Health Assessment (GN-CHA). The questionnaire, administered to 391 adults, included a list of questions on symptom frequency [4, 27]. Cluster analyses showed that symptoms of nervous system dysfunction bonded into six clusters, representing Extrapyramidal impairment, Sensory impairment, Gross motor impairment, Neuro-cognitive deficits, Cranial nerve disturbances, and Affect/Mood disorders. All clusters were related to historical indicators of Hg exposure [4].

The objective of the present study was to evaluate the 5-year persistence of reported symptoms, the progression of symptom frequency and cluster scores with respect to long-term past Hg exposure.

## Methods

### Study design

The study was carried out in partnership with Grassy Narrows First Nation, based on the OCAP® principles (Ownership, Control, Access and Possession) of First Nations information), a registered trademark of the First Nations Information Governance Centre (FNGIC) [28].

A prospective longitudinal design was used. The same questionnaire, which included a list of symptoms of nervous system dysfunction [4] was administered at Time 1 (2016–2017) and at Time 2 (2021 – 2022).

**Fig. 1:**
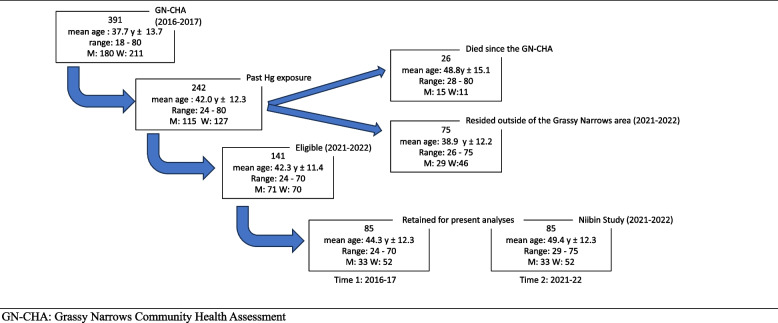
Flow chart of participant selection for follow-up study

- Time 1. The symptom questionnaire was part of the Grassy Narrows Community Health Assessment (GN-CHA), a house-to house survey of Grassy Narrows First Nation registered Band members [ 4, 27]. The survey was carried out by 9 interviewers between December 2016 and March 2017; 83.6% of the 213 houses on reserve were visited (302 adults) and 89 persons (66 households) living off-reserve were either visited or contacted by telephone, A total of 391 adult (≥ 18 years of age) Registered Grassy Narrow Band members participated in the survey.
- Time 2. The same symptom questionnaire was included in the Niibin study, carried out in July and August of 2021 and 2022, during the Covid-19 pandemic. A Covid-19 safety protocol was agreed upon with Grassy Narrows First Nation Chief and Council and followed in both summers. For the present study, inclusion criteria were persons who (i) had responded to the questionnaire in both the GN-CHA and the Niibin study, (ii) had documented past Hg exposure, and (iii) resided in Grassy Narrows or in the immediate area. Between 2017 and 2022, 26 persons (10.7%) who had participated in the GN-CHA had died. Figure 1 shows the selection process of participants from the GN-CHA to the current study. A total of 85 participants met the above criteria for a follow-up rate of 60.3%.

The questionnaire, which included socio-demographic information, life-style, past and present fish consumption and a list of symptoms, is described in detail elsewhere [4]. Symptom frequency was rated on a 5-point Likert rating scale (“never”, “rarely”, “from time to time”, “very often”, “all the time”). Higher scores on the Likert rating scale indicated greater frequency of symptoms.

### Exposure measurements

In the early 1970’s, the Medical Service Branch of Health Canada and the Ontario Ministry of Health initiated Hg biomarker testing (blood and hair) in two communities living downstream of the chloralkli plant that discharged approximately 9 metric tons of Hg into the fishing territories of Grassy Narrows and Wabaseemoong (also known as White Dog) [29]. The program continued until 1997, when average biomarker concentrations were below the Canadian guidelines for the past few years [30]. Sampling was not carried out on a regular schedule and persons sampled one year were not necessarily present the following year(s). Samples were analyzed for Hg according to the methods published by Farant [31]. MeHg in the hair samples constituted 88% of total Hg [31], mirroring the percentages reported for the fish. Walleye and Northern pike, taken from the river system downstream of the chloralkali plant, showed that MeHg made up 85-100% of total Hg concentration [32]. Studies have shown that Hg concentrations in Walleye, the fish most consumed by the people of Grassy Narrows, have remained stable since the mid-eighties to the early nineties, depending on location in this fluvial lake system [18, 19]; the biomarkers of human exposure followed the same pattern [4].

In 2019, Grassy Narrows First Nation Chief and Council obtained the community’s archived hair/blood Hg biomarker data from the historic biomonitoring programs, held by First Nations and Inuit Health Branch of the Ministry of Indigenous Services Canada, and the Ontario Ministry of Health and Long-term Care. Grassy Narrows shared this information with the research team. Most available Hg biomarker data were hair-based, and in many cases, depending on the length of hair, with values for several centimeters. From these data, we created a retrospective longitudinal year-based database for the years 1970 – 1997, with the highest measurement of hair total Hg (HHg) for each year [23]. For blood samples, Hg concentrations were converted into corresponding HHg, using a hair/blood ratio of 250 [33]. Since fish consumption varied throughout the year [22, 34], the corresponding months were also recorded and merged into low and high peak seasons for fish-eating practices. The 28-year retrospective database includes 662 persons (3639 year-based equivalent HHg measurements); the highest number of persons sampled/year (> 250) were from 1975 to 1978. Of the 662 persons included in the 1970 – 1997 database, 296 (44.7%) have since died.

### Data and statistical analyses

#### Missing values

There were few missing questionnaire symptom data (0.34% in GN-CHA and 0.73% in Niibin study); data imputation was conducted using Multivariate Imputation by using Chained Equations (MICE), relying on a set of conditional densities for each variable.

#### Symptom clusters’scores at Time 2

The algorithm developed in Time 1, which provided six symptom clusters of frequency data for 37 symptoms, was applied to the reported symptom frequencies of this study group for Time 1 and Time 2. The algorithm was based on a two-step technique that used hierarchical clustering algorithm on mixed principal component analysis (PCAMIX) in the R package ClustOfVar (for more details see Philibert et al. 2022 [4]). Symptom clusters are conventionally extracted into a single score using composite scoring, which is calculated by summing the standardized scores of each symptom belonging to the same cluster.

#### Statistical analyses

For each symptom, the McNemar test was used to compare, between Time 2 and Time 1, the combined frequencies “very often”/ “all the time” versus the lesser frequencies. A series of non-parametric paired t-tests (Wilcoxon Signed-Rank prob >|S|) assessed the difference in cluster scores between Time 2 and Time 1. Multivariate linear analyses were used to examine the contribution of age, sex, heavy drinking over the past year and smoking to the composite symptom cluster scores.

#### Mixed effects models

We chose mixed effect models (MEM) as they are robust to missing data, irregularly spaced sampling and allow for both time-invariant and time-varying covariates [35]. A series of longitudinal MEMs (LMEM) were conducted using repeated values of HHg for each year sampled; these are described in detail elsewhere [23]. Fixed and/or random effects for the following covariates were tested: year of sampling, age, sex, body mass index (BMI), attendance of residential school, level of education, current heavy drinking, current smoking. Age at sampling and sampling season were tested for random effects. Covariates were kept in the LMEM if they showed a *p*-value < = 0.10 or if they substantively altered the cluster (≥ 20% change), with the exception of age and sex, which were always maintained in the models. The normality of residuals was tested using a q-q plot. The most appropriate model was selected using the Akaike Information Criterion (AIC), the Baysian Information Criteria (BIC) and the likelihood ratio (LR) test at *p* < = 0.05.

To overcome unequal repetitions of individual HHg measurements and to reflect the time trend of long-term Hg exposure, for the longitudinal analyses, we included participants with at least 10 HHg measurements (6 men and 12 women; median age: 57 years) for a total of 239 HHg values, covering a period from 10 to 21 years between 1970 and 1997. To ensure that there were sufficient observations for the analyses, we estimated the minimal required sample size, based on formulas from Hedeker and co-authors [36] and direct calculations using the G*power software [37–40]. Since one centimeter of hair represents an accumulation of Hg during approximately one month [41], we used a low correlation of repeated measures between yearly-based samples. Because the effect size was unknown, 0.20 was chosen. Power analyses were set at 80%, with a two-tailed 5% hypothesis test; the minimal number of participants required for 10 repeated measurements was between 14 and 22.

Although the longitudinal analyses respected the sample size estimation and power analyses, the number of persons with at least 10 HHg year-based measurements over this sampling time period was limited to 18. To support our findings, a series of sensitivity analyses were conducted with a lower requirement for repeated HHg measurements (at least 5 year-based HHg), thus increasing the sample size.

Covariates (socio-demographic characteristics and lifestyle), collected at Time 1 and Time 2, were used for statistical analyses, when appropriate.

#### Statistical packages

Database management, descriptive analyses, paired analyses, and Bowker-McNemar statistical tests were performed using JMP Professional 17·0 Statistical Analysis Hardware (SAS Institute). The R statistical computing software version 3·6·1 (R Core Team, 2016) was used for imputation of missing data and for deriving composite scores from clustering (the R package Mice and ClustOfVar, respectively). The LMEM were conducted with Stata 16 Software (StataCorp. 2019. Stata Statistical Software: Release 16·0 College Station, TX: Stata Corporation). MEM and LMEM results in Stata were compared with those found in the R package lme4.

## Results

The 85 participants included 53 women (62.4%) and 32 men (37.6%). Their median age at Time 2 was 47 years old (IQR 40 – 59y, ranging from 29 -75y), with no difference in age distribution between women and men. Almost all participants had attended primary school (98.8%) and 63 (74.1%) had at least some high school. Over half (52.9%) had furthered their education through various training programs.

Over the 5-year period, there was an increase in the proportion of retirees and/or disabled persons (11.8% to 20.0%; McNemar-Bowker Chi square = 3.77; *p* = 0.052). The median age of those who, at Time 2, were retired (*n* = 5) or disabled (*n* = 11) was 59 years of age, ranging from 36 to 74y. Between the two periods, there was no difference in the proportion of heavy drinkers, as defined in the First Nation Regional Health Survey 2008/2010: 5 drinks or more in one drinking occasion at least once/month in the previous year [42] (39.2% vs 35.4%; McNemar-Bowker Chi square = 0.47; *p* = 0.491), but more persons reported currently smoking (36.5% vs 48.2%; McNemar-Bowker Chi square = 5.55; *p* = 0.018). The prevalence of current smokers and heavy drinkers significantly decreased with age.

Over the 5-year period, there was a downward shift in participants’ perception of their overall health. Proportionally more persons reported their health as fair at Time 2 compared to Time 1 (50.6% vs 30.1%; McNemar-Bowker Chi square = 9.32; *p* = 0.002) and poor (20.2% vs. 11.9%; McNemar-Bowker Chi square = 2.88; *p* < 0.090).

Grouped by Cluster, Table 1 presents, for each symptom, the prevalence of persons who reported its frequency as “very often” or “all the time” at Time 1 and Time 2. Table 1 likewise contains the results of paired analyses for each symptom; the increase or decrease between Time 1 and Time 2 for those who reported “very often”/ “all the time” are presented. For the large majority of symptoms, there was a significant increase in reported symptom frequency at Time 2. The most striking increases were for dropping things, tingling in hands and symptoms of gross motor impairment, neurocognition, mood, speech and sleeping difficulties. At Time 2, over 40% of participants reported dropping things, tingling sensations in their hands, trouble lifting and climbing stairs, forgetting where one has put things, apathy (feeling like doing nothing), waking up at night and tiredness. Figure S1 provides a graphic illustration of the distribution of reported frequencies for each symptom for “never/rarely”, “from time to time”, “very often” and “all the time” for Time 1 (Figure S1a) and Time 2 (Figure S1b).

**Table 1:** Distribution of symptoms, reported “very often” or “all the time” at Time 1 and Time 2

**Symptoms within clusters**	**Prevalence of reporting “very often” and “all the time”**		**Paired increase and decrease between** **Time 1 to Time 2**		**McNemar- Bowker** **Chi square**
	Time 1 n (%)	Time 2 n (%)	Increase n (%)	Decrease n (%)	
**Cluster 1** **Extrapyramidal impairment**					
Pain in legs	17 (20.0%)	30 (35.3%)	22 (25.9%)	9 (10.6%)	5.45*
Stiff shoulders	15 (17.7%)	26 (30.6%)	17 (20.0%)	6 (7.06%)	5.26*
Pain in arms	11 (12.9%)	19 (22.4%)	16 (18.8%)	8 (9.41%)	2.67
Burning feet	16 (18.2%)	27 (31.8%)	16 (18.8%)	5 (5.88%)	5.76*
Drop things	13 (15.3%)	35 (41.2%)	26 (30.6%)	4 (4.71%)	16.13***
Stumble	11 (12.9%)	26 (30.6%)	19 (22.4%)	4 (4.71%)	9.78**
Fall	5 (5.88%)	11 (12.9%)	10 (11.8%)	4 (4.71%)	2.57
Feet tremors	8 (15.2%)	21 (24.7%)	17 (20.0%)	4 (4.71%)	8.05**
Stop or freeze	3 (3.53%)	9 (10.6%)	8 (9.41%)	2 (2.35%)	3.60
**Cluster 2** **Sensory impairment**					
vNumbness in hands	14 (16.5%)	31 (36.5%)	22 (25.9%)	5 (5.88%)	10.70**
Tingling in feet	10 (11.8%)	30 (35.3%)	25 (29.4%)	5 (5.88%)	13.33***
Numbness in feet	11 (12.9%)	27 (31.8%)	21 (24.7%)	5 (5.88%)	9.88**
Tingling in hands	13 (15.3%)	36 (42.4%)	28 (32.9%)	5 (5.88%)	16.03***
Dullness in feet	10 (11.8%)	19 (22.4%)	12 (14.1%)	3 (3.53%)	5.40*
Dullness in hands	9 (10.6%)	15 (17.6%)	10 (11.8%)	4 (4.71%)	2.57
**Cluster 3** **Cranial disturbances**					
Difficulty swallowing	5 (5.88%)	24 (28.2%)	22 (25.9%)	3 (3.53%)	14.44***
Loss of smell	8 (9.41%)	12 (14.1%)	8 (9.41%)	4 (4.71%)	1.33
Loss of taste	6 (7.06%)	0	0	6 (7.06%)	N/A
Tingling around mouth	7 (8.24%)	6 (7.06%)	5 (5.88%)	6 (7.06%)	0.09
Choking	2 (2.35%)	15 (17.6%)	14 (16.5%)	1 (1.18%)	11.27***
**Cluster 4** **Gross motor impairment**					
Trouble lifting	19 (22.4%)	53 (62.4%)	37 (43.5%)	3 (3.53%)	28.90***
Difficulty climbing stairs	12 (14.1%)	36 (42.4%)	25 (19.4%)	1 (1.18%)	21.15***
Difficulty walking 5 min without resting	9 (10.6%)	29 (34.1%)	23 (27.1%)	3 (3.53%)	15.38***
Trouble lifting 10 lbs	14 (16.5%)	28 (32.9%)	19 (22.4%)	2 (2.35%)	13.76***
**Cluster 5** **Neuro-cognitive deficits**					
Forget where to put things	14 (16.5%)	46 (54.1%)	36 (42.4%)	1 (1.18%)	33.11***
Forget doing things	7 (8.24%)	27 (31.8%)	21 (24.7%)	1 (1.18%)	18.18***
Trouble hearing	14 (16.5%)	30 (35.3%)	19 (22.4%)	3 (3.53%)	11.64**
Difficulty pronouncing words	7 (8.24%)	28 (32.9%)	23 (27.1%)	2 (2.35%)	17.64***
Difficulty having speech understood	5 (5.88%)	33 (38.8%)	30 (35.3%)	2 (2.35%)	24.50***
**Cluster 6** **Affect/mood disorders**					
Wake up at night	33 (38.8%)	69 (81.2%)	41 (48.2%)	5 (5.88%)	28.17***
Trouble falling asleep	27 (31.8%)	44 (51.8%)	26 (30.6%)	9 (10.6%)	8.26**
Tired	21 (24.7%)	47 (55.3%)	32 (37.7%)	6 (7.06%)	17.79***
Anxious	8 (9.41%)	30 (35.3%)	22 (25.9%)	5 (5.88%)	10.70**
Difficulty concentrating	14 (16.5%)	26 (30.6%)	20 (23.5%)	6 (7.06%)	7.54**
Doing nothing	10 (11.8%)	46 (54.1%)	41 (48.2%)	5 (5.88%)	28.17***
Depressed	9 (10.6%)	19 (22.4%)	15 (17.7%)	5 (5.88%)	5.00*
Irritable	8 (9.41%)	30 (35.3%)	27 (31.8%)	5 (5.88%)	15.13***

^*^ *p* < 0.05; ^**^ *p* < 0.01; ^***^ *p* < 0.001 N/A non-applicable

Graphic representations of the means of the frequency scores for each symptom with respect (< and ≥ 50 years at Time 2), are presented in Figures S2(a) and S2(b). At Time 1 (Figure S2(a)), the younger persons (< 45 years old), had lower symptom frequency scores compared to the older persons, with the exception of stop or freeze, numbness in hands, choking, difficulty swallowing and most symptoms of the cluster Affect/Disorders, which are similar. Five years later (Figure S2b), for almost all symptoms, there is an increase in reported frequency for both the older and younger participants.

Figure S3 shows the mean and standard error of the paired differences from Time 1 to Time 2 for the younger and older participants; significant differences at *p* < 0.05 (Wilcoxon Signed Rank for Test Mean = 0) are indicated. For most symptoms, reported frequency significantly increased over the 5-year period for both the older and younger participants, with a mean increase of approximately 1 point on the Likert scale. Sensory impairment shows a different pattern, with pain in legs significantly increasing for the older persons, while tingling and numbness in feet and hands increased significantly for the younger persons. The mean paired difference in reported frequency of loss of taste at Time 1 was significantly higher than at Time 2. It should be noted that very few persons reported this symptom “very often” and “all the time” (see Table 1).

Table 2 presents the descriptive analyses of cluster scores at Time 1 and Time 2. Higher cluster scores indicate higher symptom frequencies. Paired analyses showed a highly significant increase over the 5-year period for all clusters.

**Table 2:** Cluster scores’ distribution and paired analyses between Time 1 and Time 2 (*n* = 85)

	Time 1			Time 2		
	Median	IQR	Median	IQR	Wilcoxon Signed Rank (S)	p
Cluster 1 (Extrapyramidal impairment)	5.06	(3.39 – 7.26)	6.22	(4.58 – 8.23)	814	< 0.0001
Cluster 2 (Sensory impairment)	5.21	(2.94 – 6.58)	6.24	(4.51 – 7.44)	871	< 0.0001
Cluster 3 (Cranial nerve disturbances)	5.05	(3.60 – 6.30)	5.60	(4.11 – 6.58)	607	0.0036
Cluster 4 (Gross motor impairment)	4.57	(3.55 – 6.36)	6.65	(4.57 – 8.50)	1238	< 0.0001
Cluster 5 (Neuro-cognitive deficits)	4.80	(3.51 – 6.00)	6.86	(5.48 – 8.01)	1551	< 0.0001
Cluster 6 (Affect/Mood disorders)	5.04	(3.00 – 6.45)	6.55	(5.20 – 7.74)	1212	< 0.0001

Multivariate regression analyses were performed to examine cluster scores at Time 1 and Time 2 with respect to the variables age, sex, heavy drinking and smoking. For Time 1, age significantly contributed to the scores for Clusters 1–5. For Cluster 6 (Affect/Mood disorders), no association was observed with age. Men and women presented similar scores for all clusters, with the exception of Cluster 6 (Mood/Affect Disorders) and Cluster 4 (Gross motor impairment), where women tended to report higher scores compared to men (*p* = 0.064 and *p* = 0.109, respectively). A positive association was observed with heavy drinking over the year prior to questionnaire administration for Sensory Impairment (*p* = 0.033) and Affect/Mood Disorders (*p* = 0.027) at Time 1. For Time 2, age was significantly associated with Clusters 3–5 (Cranial Nerve Disturbances (*p* = 0.044), Gross Motor Impairment (*p* < 0.0001) and Neurocognitive Deficits (*p* = 0.018), while Cluster 1 (Extrapyramidal Impairment) showed a tendency (*p* = 0.069). In the model for Cluster 4 (Gross Motor Impairment), women presented significantly higher scores compared to men (*p* = 0.001). No significant associations were observed for heavy drinking over the past year and current smoking.

For the difference (Time 2 – Time 1) in symptom frequency reporting, the only significant association was a negative relation with age for Cluster 2 (Sensory impairment (F = 8.83; *p* = 0.004), reflecting the greater increase in symptom frequency for some of the symptoms within this Cluster in younger persons (see Figure S3).

**Fig. 2:**
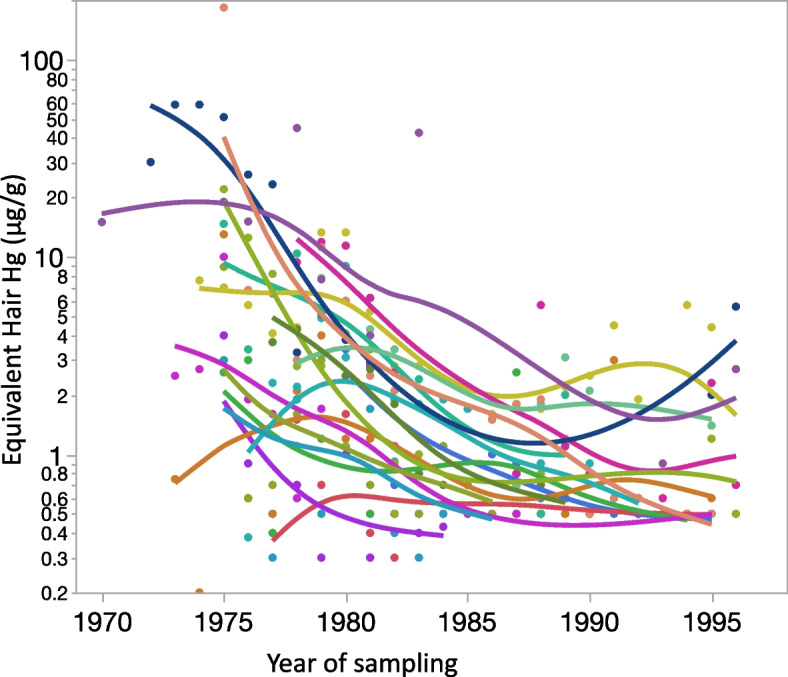
Distribution of year-based log hair Hg equivalent for persons with at least 10 measurements

For LMEMs, persons with at least 10 repeated year-based HHg measurements between 1970 and 1997 (*n* = 18; 12 women, 6 men) were retained. Figure 2 shows their year-based HHg between 1970 and 1997. During this period, there was an overall decrease in HHg over time, with important inter-individual variations.

The selected sub-group with at least 10 HHg measurements, was significantly older than those who had fewer HHg measurements (median 57y, (IQR: 51 – 65y, ranging from 41 to 74y) vs. median 44 y (IQR: 38 – 56y, ranging from 29 -75y); Wilcoxon Signed Rank Chi square = 11.2; *p* < 0.001), while the proportion of men and women was similar. Comparison of those who were retained for the ≥ 10HHg sub-group with respect to the others, showed no difference for all cluster scores at Time 1. At Time 2, Cluster 3 (Cranial disturbances) and Cluster 4 (Gross motor impairment) were higher in the ≥ 10HHg sub-group compared to the others (Wilcoxon Signed Rank Chi square = 5.47; *p* = 0.020 and Wilcoxon Signed Rank Chi square = 8.02; *p* = 0.005, respectively). Over the 5-year period, only the score for Cluster 4 (Gross motor impairment) increased significantly more rapidly in the retained ≥ 10HHg sub-group compared to the others (Wilcoxon Signed Rank Chi square = 4.05; *p* = 0.040).

Table 3 shows the results of LMEM associations between long-term past HHg and the symptom cluster scores at Time 1 and Time 2 for the ≥ 10HHg sub-group. At Time 1, long-term exposure was associated with cluster scores for Cluster 1 (Extrapyramidal impairment) and Cluster 5 (Neuro-cognitive deficits). A tendency was observed for Cluster 6 (Affect/Mood disorders). At Time 2 long-term exposure was significantly associated with all cluster scores, with the exception of Cluster 2 (Sensory impairment).

**Table 3:** Longitudinal Mixed Effect Model estimates for persons with ≥ 10 repeated hair Hg (independent variable) with cluster scores at Time 1 and 2 (dependent variables)

	Time 1				Time 2			
	N	Estimate	95% CI	*p*-value	N	Estimate	95% CI	*p*-value
Cluster 1 (Extrapyramidal impairment)	14	0.37	0.02 – 0.72	0.038	15	1.32	0.03 – 2.62	0.044
Cluster 2 (Sensory impairment)	15	0.28	-0.03 – 0.57	0.074	15	-0.09	-0.32 – 0.15	0.480
Cluster 3 (Cranial nerve disturbances)	14	-0.01	-0.54 – 0.53	0.984	17	0.63	0.22 –0.71	0.032
Cluster 4 (Gross motor impairment)	14	0.09	-0.41 – 0.24	0.594	16	1.16	0.44 –1.89	0.002
Cluster 5 (Neuro-cognitive deficits)	14	0.57	0.26 – 0.88	0.000	16	1.24	0.41 – 2.07	0.003
Cluster 6 (Affect/Mood disorders)	14	0.39	-0.01 – 0.79	0.057	17	0.66	0.27 – 1.06	0.001

In all longitudinal effects mixed models, age, sex, time, sampling season and year of sampling were included as fixed factors; age of sampling is nested in year of sampling as random factor. Outliers were excluded based on heteroskedasticity of residuals

*CI* Confidence Interval

Table 4 presents the associations with past HHg, for the ≥ 10HHg sub-group, for the score difference for each cluster (Time 2 – Time 1). The differences between Time 2 and Time 1 increased with long-term past HHg concentrations for all clusters.

**Table 4:** Longitudinal Mixed Effect Model estimates for ≥ 10 repeated hair Hg (independent variable) with cluster differences (Time 2 – Time 1) (dependent variables)

**Composite symptom cluster variable** **Time 2-Time 1**	N	Estimate	95% CI	p
Cluster 1 (Extrapyramidal impairment)	18	1.28	0.78 – 1.77	0.000
Cluster 2 (Sensory impairment)	18	1.37	0.86 – 1.88	0.000
Cluster 3 (Cranial nerve disturbances)	18	1.06	0.50 – 1.63	0.000
Cluster 4 (Gross motor impairment)	18	0.41	0.12 – 0.70	0.006
Cluster 5 (Neuro-cognitive deficits)	18	1.53	0.80 – 2.25	0.000
Cluster 6 (Affect/Mood disorders)	18	0.69	0.32 – 1.05	0.000

In all models, age, sex, time, sampling season and year of sampling were included as fixed factors; age of sampling is nested in year of sampling as random factor. Based on heteroskedasticityof residuals, there were no outliers

*CI* Confidence Interval

Although persons with 10 – 21 year-based HHg measurements over the 28-year period provided a good portrait of overall exposure, there were only 18 persons in this sub-group. We thus carried out LMEMs for persons with at least 5 year-based HHg measurements (*n* = 39); their median age at Time 2 was 59y; (IQR: 54 – 65y; range: 41 – 75y), although power calculations showed that at least 42 persons were required for acceptable LMEM analyses. The results, which are similar to the above, are presented in Tables S1 and S2.

## Discussion

In the present study, the symptom clusters had been constructed and validated, using the database of 391 persons from the Grassy Narrows First Nation Community Health Assessment (GN-CHA), carried out in 2016- 2017 [4]. These clusters reflect the multifaceted outcomes of Hg-related neurological deficits [8, 43–47] and were shown to be associated with different aspects of this community’s past Hg exposure [4]. The present follow-up, carried out in 2021–2022 (Time 2) focussed on 85 GN-CHA participants with documented Hg exposure between 1970 and 1997. Over the 5-year period, there was an increase in self-reported frequency for a large majority of individual symptoms, as well their overall composite cluster scores. For a sub-group of persons with 10—21 year-based HHg measurements, born between 1947 and 1980, longitudinal age-adjusted analyses showed that the increase in all cluster score differences (Time 2 – Time 1) was associated with higher past HHg exposure, suggesting a faster rate of decline of nervous system functions.

In the present study, reported frequency for most symptoms and cluster scores at Time 1 (2016–2017) and Time 2 (2021–2022) increased with age. Age differences in reporting specific and non-specific symptoms of Minamata Disease in Grassy Narrows First Nation community was first noted by Takaoka and co-authors [48]. In 2010, these authors administered a symptom questionnaire to 80 volunteers from Grassy Narrows and 88 randomly chosen residents from the Minamata area (Japan), who had been exposed to high levels of MeHg through fish consumption prior to 1968, as well as to a Japanese reference group [48]. The authors reported, that for most symptoms, the prevalence for older persons from Grassy Narrows (46 – 74 years of age), who reported the frequency as “always” was consistently higher compared to the younger persons and similar to the Minamata residents [48].

It is noteworthy that, like in the study by Takaoka and colleagues [48], the “older” persons in the present study were not very old. Indeed, at the beginning of the 5-year follow-up, the “older persons” were between 45 and 70 years of age, with half between 50 and 60 years. While both older and younger participants showed an increase in symptom frequency over the 5-year period, for the older group, the pattern of symptom frequency distribution at the two times was similar. For the younger group, there appears to be a change over time; at Time 2, the pattern of symptom frequencies bears more resemblance to that of the older participants. Interestingly, almost all symptoms of sensory impairment increased significantly in the younger persons, but not in the older persons. This may be because persons in the older group were higher on the Likert scale at Time 1 and the frequency did not increase as much, or that some older persons no longer felt the tingling and/or numbness in their extremities. Peripheral somatosensory loss is a recognizied sign of mercury poisoning [11, 49–51], however, other factors, such as diabetes, may also contribute [52].

At Time 1 and Time 2, all of the cluster scores increased with age, with the exception of Cluster 6 (Affect/Mood Disorders), similar to what had been previously observed in this population [4]. Age, however, did not contribute to the increase in any of the cluster scores between Time 1 and Time 2, with the exception of the Sensory Impairment cluster, where an inverse association, probably due the factors described above, was observed.

Symptom cluster scores were similar for men and women, with the exception of gross motor functions, where women reported higher frequencies at both Time 1 and Time 2. Gender differences in gross motor impairment may reflect over-all strength, other health conditions and/or attitude [53, 54]. There was no difference in the increase in symptom cluster scores over the 5-year period, between men and women, for any of the clusters.

The findings of the present study further raise the question of the possible progressive nature of Hg poisoning in this community. In a previous study in Grassy Narrows, Harada and co-workers examined 27 persons in 1975 and again in 2002/2004, when Hg exposure was considerably lower [24–26]. Those with mild or almost no symptoms in the first examination showed typical symptoms of Minamata Disease some 25 years later. Symptoms had sufficiently progressed for 13 persons to receive a diagnosis of Minamata Disease and 11, a diagnosis of Minamata Disease with complications. There were marked increases in sensory disturbances, ataxia, visual field constriction, speech impairment, imbalance and tremor. The five-year progression in self-reported symptom clusters in the present study, and their association with past Hg exposure for persons > 40 years of age, is concerning.

In the present study, we did not have information on symptom onset. Several authors have suggested that there may be a lag time or latent phase for MeHg neurotoxicity [55–58]. Weiss and co-authors [57] suggest that longer dose-dependent latency periods, associated with low-level chronic MeHg exposure, may be attributed to cell loss due to the combined effects of toxicity and aging. In a recent review, Branco and co-authors [59] noted that even at low levels of exposure, Hg neurotoxicity appears to be a multifactorial process due to the interaction between different forms of Hg within the brain, which could trigger cellular events well before symptoms are manifest. In a study of the onset of neurologic symptoms among residents in the polluted areas in and around Minamata, Japan, the onset of neurologic symptoms grouped into 5 clinical categories (cramps, numbness, stumbling tendency, difficulty in fine finger tasks, and limited vision), began, on average, more than 10 years after the factory stopped releasing MeHg into Minamata Bay [3].

Although the Likert scale, used in the CHA and Niibin questionnaires, provided a convenient tool to build composite cluster scores, the transformation of qualitative responses into an ordinal scale is susceptible to bias and information loss since there is an assumption of equidistance between scale units [60]. Indeed, the intervals between “never” and “rarely” or “very often” and “all the time” do not necessarily reflect the same distance. The clustering approach, based on constructs for co-occuring symptom frequencies, provided a continuous composite score, which could then be analysed over time and with respect to Hg exposure and other covariates.

Self-reported symptoms may also be a source of information bias [61]. In the present study, a series of indices were used to ensure consistency and reliability within the symptom clusters. The Cronbach alpha (0.82—0.94) confirmed internal consistency and reliability and the goodness of fit of Confirmatory Factor analyses validated the constructs [4]. The alphas are similar to those reported in a study using standardized questionnaires with Native American and Alaskan communities; the authors of that study noted that the Cronbach alpha for this population (> 0.87) exceeded published data for the general population [62]. In the present study, there was a large intra-individual variation in the reported frequency for the different symptoms. There was also a large variation in the distribution prevalence of the Likert scale between symptoms. These patterns reflect the importance that the people of Grassy Narrows, who have fought for 50 years for recognition of the impact of Hg on their health, give to providing trustworthy answers to support sound investigations of how Hg has affected their health. They also reflect Grassy Narrows’ Anishinaabe culture which values honesty [63, 64]. Previous studies with Grassy Narrows First Nation have validated self-reports on childhood fish consumption and measured HHg between 10 and 15 years of age [4, 27].

There are several limitations to the history of past Hg exposure, notably inconsistent sampling and the absence of data for the past 20 years. Although the retrospective HHg biomarker database, derived from a government biomonitoring program [22], spanned 28 years of exposure, sampling was irregular, with non-uniform intervals between successive measurements. Moreover, no information was available on the variations associated with changing Hg ingestion and excretion [65], To compensate for the longitudinal unevenness of sampling time intervals, we extracted a sub-group with ≥ 10 year-based HHg measurements to reflect the time trend of exposure, and included sampling season to take into account monthly variations in fish consumption. Although this small sub-group (*n* = 18) respected the required criteria for sample size estimation and power analyses, we likewise tested longitudinal associations with a larger sub-group by including those with ≥ 5 year-based HHg measurements. While this had the advantage of conducting analyses on twice as many persons (*n* = 39) of similar age distribution (41 – 75y), it was less representative of the entire exposure period, and the sample size was slightly below the minimum requirement for power and reliability of results. Despite these limitations, the results for the larger group were similar to those with ≥ 10 HHg measurements, providing support for our findings.

The biomarker program was halted in 1997, when few persons surpassed the Canadian guidelines for the “normal acceptable range” for hair Hg: 6—30 µg/g [66, 67]. For participants in the present study, median HHg was below 1 µg/g from 1995 – 1997. In 2003, hair Hg concentrations were measured in 87 people from Grassy Narrows; mean hair Hg was 1.3 µg/g ± 1.8 µg/g, ranging from non-detectable to 7.4 µg/g [68]. In a second study, carried out in Grassy Narrows in 2011, median hair Hg was 0. 71 µg/g (IQR: 0.29 – 1.38 µg/g) for 21 persons (Chan, H.M. personal communication). In the 2021–2022 Niibin study, blood Hg was assessed in 117 persons; median equivalent HHg was 0.8 µg/g (IQR: 0. 20 – 1.83 µg/g) [69]. Although sparse, these data suggest that for the past 20 years exposure was relatively stable. However, we cannot rule out the contribution of exposure over the past two decades, or the contribution of prenatal exposure to symptom evolution.

A further limitation to the present study is the loss to follow-up and sample size. The GN-CHA was a house-to-house survey, carried out in winter months when more people remain on reserve. The follow-up study was based on the selection of GN-CHA participants with documented Hg exposure, who lived in Grassy Narrows or the nearby region. Coupled to the proportion of deaths since the GN-CHA (10.7%), 141 persons (36%) met the eligibility criteria. The follow-up rate of those who were eligible (60%) is at the lower end of acceptability [70]. Follow-up was carried out in the summers of 2021 and 2022 and several events may have played a role in persons not returning. The first year of follow-up (2021) was during the Covid-19 pandemic and several persons, who were invited to participate, preferred not to come or were ill. In the second year, during the recruitment period, the community was evacuated because of forest fires and flooding. Moreover, it was the first summer following confinement due to the pandemic and many men, were working outside of the community, notably as firefighters, which may account for the lower follow-up rate in men compared to women.

The context of Hg exposure in Grassy Narrows is complex. Although the discharge began in 1962, it was not until 1970 that very high concentrations of Hg in fish in the rivers and lakes near their reserve were reported [32], and the public and the community began to be informed. During that period, most families ate fish every day [71]. Fish guiding for nearby lodges was common employment for men from almost every family, while women worked at the lodges in housekeeping and kitchens [71]. Biomarker data showed three stages of exposure: the highest was between 1970 and 1977, declining until 1987, and stabilizing in the 1990’s [23]. The decrease in Hg exposure reflected the decrease in fish Hg concentrations, coupled to a growing awareness of Hg toxicity and a decrease in fish consumption [19, 30]. Despite the very high exposure over the many years following the discharge and the reported cases of Minamata Disease [24, 25], the possible impact of long-term exposure in this community has rarely been addressed.

The findings of this study raise the issue of follow-up and adequate health care for this and other communities with previous high Hg exposure. In 1989, Postl and co-authors [72] reported that in Grassy Narrows First Nation there was higher morbidity and mortality compared to district, provincial and national standards. The authors noted the inadequancy of health services and recommended the establishment of an elders home which should serve as the focus for an expanded health center [72]. It was not until 2017 that the federal government committed to support Grassy Narrows’ demand for a health center that would provide care and services for persons suffering from Hg poisoning. When writing this (December, 2023), construction of the Mercury Care Home and Wellness Centre had not yet started and there had not been a physician in the community for a period of 17 months (Marshall, L-A. personal communication).

Although the Canadian Hg biomonitoring program, initiated in the early seventies, revealed that exposures in Grassy Narrows were higher than in other First Nation communities [22], high blood Hg concentrations (≥ 20 µg/L) were likewise observed in many other First Nation communities [73]. Although for these communities, current Hg exposure does not necessarily present a clinical health risk [67], the findings of this study suggest that past long-term high Hg exposure may contribute to current and possibly future poor health.

## Conclusions

Sixty years after the beginning of the discharge and 20 years after mean HHg concentrations were below Canadian guidelines, long-term past high Hg exposure for persons > 40 years of age was significantly associated with an increase in symptom frequency, over a short 5-year period, within all of the clusters of nervous system dysfunction. The increase in reported symptom frequency over the 5-year period observed for younger persons needs to be adequately monitored and treated.

### Supplementary Information

Supplementary Material 1.

## Data Availability

Restrictions apply to the availability of these data. Data were obtained from and are the property of the Grassy Narrows First Nation, in keeping with the First Nations principles of Ownership, Control, Access, and Possession (OCAP). Any request for the data should be addressed to Grassy Narrows First Nations.
