# Supplementary material for: The evolution of symptoms of nervous system dysfunction in a First Nation community with a history of mercury exposure: a longitudinal study
Source: Environ Health. 2024 May 31;23:50. doi: 10.1186/s12940-024-01089-9 (PMC11140928; doi:10.1186/s12940-024-01089-9)
Supplement: Supplementary file 1 — Supplementary Material 1. [file 12940_2024_1089_MOESM1_ESM.docx]

**Supplemental Information**

The evolution of symptoms of nervous system dysfunction in a First Nation community with a history of mercury exposure: a longitudinal study

**Table of contents**

**Table S1.** Longitudinal Mixed Effect Model estimates for persons with ≥5 repeated hair Hg (independent variable) with cluster scores at Time 1 and 2 (dependent variables)

**Table S2.** Longitudinal Mixed Effect Model estimates for ≥5 repeated hair Hg (independent variable) with cluster differences (Time 2 – Time 1) (dependent variables)

**Figure S1a**. Frequency distribution of self-reported symptoms in Time 1 (2016-2017)

**Figure S1b.** Frequency distribution of self-reported symptoms in Time 2 (2021-2022)

**Figures S2a and S2b** Mean frequency distribution of self-reported symptoms for each cluster in Time 1 (2016-2017) (S2a) and Time 2 (2021-2022) (S2b), by age category

**Figure S3.** Mean paired difference and standard errors of the frequency of self-reported symptoms from Time 1 (2016-2017) to Time 2 (2021-2022)

**Table S1.** Longitudinal Mixed Effect Model estimates for persons with ≥5 repeated hair Hg (independent variable) with cluster scores at Time 1 and 2 (dependent variables)

|  | Time 1 | | | | | Time 2 | | | |
| --- | --- | --- | --- | --- | --- | --- | --- | --- | --- |
|  | N | Estimate | | % CI | p | N | Estimate | % CI | p |
| Cluster 1  (Extrapyramidal impairment) | 38 | 0.38 | 0.20 – 0.56 | | 0.000 | 38 | 0.38 | 0.15 – 0.61 | 0.001 |
| Cluster 2  (Sensory impairment) | 38 | 0.57 | 0.30 – 0.83 | | 0.000 | 38 | 0.59 | 0.34 – 0.84 | 0.000 |
| Cluster 3  (Cranial nerve disturbances) | 38 | 0.51 | 0.19 – 0.84 | | 0.002 | 36 | 0.30 | 0.05 – 0.57 | 0.019 |
| Cluster 4  (Gross motor impairment) | 39 | 0.27 | 0.20 – 0.52 | | 0.031 | 34 | 0.29 | 0.04 – 0.55 | 0.025 |
| Cluster 5  (Neuro-cognitive deficits) | 39 | 0.41 | 0.13 – 0.69 | | 0.004 | 37 | 0.16 | 0.01 – 0.33 | 0.057 |
| Cluster 6  (Affect/Mood disorders) | 39 | 0.40 | 0.21 – 0.59 | | 0.000 | 38 | 0.44 | 0.15 – 0.72 | 0.003 |

In all longitudinal effects mixed models, age, sex, sampling season and year of sampling were included as fixed factors; age of sampling is nested in year of sampling as random factor. Outliers were excluded based on heteroskedasticity of residuals.

CI = Confidence Interval

**Table S2.** Longitudinal Mixed Effect Model estimates for ≥5 repeated hair Hg (independent variable) with cluster differences (Time 2 – Time 1) (dependent variables)

| **Composite symptom cluster variable**  **Time 2-Time 1** | N | Estimate | | % CI | p |
| --- | --- | --- | --- | --- | --- |
| Cluster 1  (Extrapyramidal impairment) | 37 | 0.27 | 0.07 – 0.48 | | 0.010 |
| Cluster 2  (Sensory impairment) | 36 | 0.32 | 0.09 – 0.56 | | 0.006 |
| Cluster 3  (Cranial nerve disturbances) | 39 | 0.33 | 0.03 – 0.63 | | 0.028 |
| Cluster 4  (Gross motor impairment) | 38 | 0.30 | 0.12 – 0.47 | | 0.054 |
| Cluster 5  (Neuro-cognitive deficits) | 39 | 0.29 | 0.07 – 0.50 | | 0.009 |
| Cluster 6  (Affect/Mood disorders) | 39 | 0.44 | 0.26 – 0.61 | | 0.000 |

In all models, age, sex, sampling season and year of sampling were included as fixed factors; age of sampling is nested in year of sampling as random factor. Based on heteroskedasticity of residuals, there were no outliers.

CI = Confidence Interval

Figure S1a. Frequency distribution of self-reported symptoms in Time 1 (2016-2017)

Figure S1b. Frequency distribution of self-reported symptoms in Time 2 (2021-2022)

**Figures S2a and S2b.** Mean frequency distribution of self-reported symptoms for each cluster in Time 1 (2016-2017) (S2a) and Time 2 (2021-2022) (S2b), by age category

Figure S3. Mean paired difference and standard errors of the frequency of self-reported symptoms from Time 1 (2016-2017) to Time 2 (2021-2022)
